# Supplementary material for: Preserved sensory processing but hampered conflict detection when stimulus input is task-irrelevant
Source: eLife. 2021 Jun 14;10:e64431. doi: 10.7554/eLife.64431 (PMC8294845; doi:10.7554/eLife.64431)
Supplement: Figure 2—source data 1. [file elife-64431-fig2-data1.zip › Figure 2/Figure 2 source data 2.rtf]

Source files for behavioural results of experiment 1 (related to Figure 2A)-------------------------------------------------------------In Figure 2A, the conflict effect (incongruent - congruent) is shown for reaction times (RT) and error rates (ER). These data are plotted for both behavioural tasks of Experiment 1. The data shown here were calculated across experimental sessions (excluding training sessions).Figure2A_behavioral results is a csv table containing the data used for Figure 2A Rows represent single subjects.Code          			Explanation----          				-----------conflict_effect_RT_CDI      Conflict effect in reaction for content discrimination task Iconflict_effect_ER_CDI     Conflict effect in error rates for content discrimination task Iconflict_effect_RT_VRDM 	Conflict effect in reaction times for vertical RDM taskconflict_effect_ER_VRDM	Conflict effect in error rates for vertical RDM task
