# Supplementary material for: Preserved sensory processing but hampered conflict detection when stimulus input is task-irrelevant
Source: eLife. 2021 Jun 14;10:e64431. doi: 10.7554/eLife.64431 (PMC8294845; doi:10.7554/eLife.64431)
Supplement: Figure 2—figure supplement 1—source data 1. [file elife-64431-fig2-figsupp1-data1.zip › Figure 2 - Supplement 1/Figure 2 ΓÇô Supplement 1 source data 3.rtf]

Source files for behavioural results of Experiment 1 (related to Figure 2 - Supplement 1)-------------------------------------------------------------In Figure 2 - Supplement 1A-B, the conflict effect (incongruent - congruent) is shown for reaction times (RT) and error rates (ER). These data are plotted for content discrimination task I of Experiment 1. The data is shown for separate experimental sessions. Figure 2 - Supplement 1 source data 1 is a csv table containing the raw data used for Figure 2 - Supplement 1A-BRows represent single subjects.Code          			Explanation----          				-----------CD_RT_CE_1 			Conflict effect in reaction time for session 1 of content discrimination task ICD_RT_CE_2			Conflict effect in reaction time for session 2 of content discrimination task ICD_ER_CE_1 			Conflict effect in error rate for session 1 of content discrimination task ICD_ER_CE_2 			Conflict effect in error rate for session 2 of content discrimination task IFigure 2 - Supplement 1 source data 2 is a csv table containing the statistical results that are shown in for Figure 2 - Supplement 1A-BFor every session of content discrimination task I, we performed a one sample t-test on the conflict effect (incongruent - congruent) against zero for RT and ER.Code          			Explanation----          				-----------Session				Experimental session of content discrimination task Idv					dependent variable: either RT or ERt					student-t statisticdf					degrees of freedomp					p-valueCohen’s d				effect size in Cohen’s d	
