# Supplementary material for: Preserved sensory processing but hampered conflict detection when stimulus input is task-irrelevant
Source: eLife. 2021 Jun 14;10:e64431. doi: 10.7554/eLife.64431 (PMC8294845; doi:10.7554/eLife.64431)
Supplement: Figure 4—source data 1. [file elife-64431-fig4-data1.zip › Figure 4/Figure 4 source data 2.rtf]

Source files for behavioural results of experiment 2 (related to Figure 4A)-------------------------------------------------------------In Figure 4A, the conflict effect (incongruent - congruent) is shown for reaction times (RT) and error rates (ER). These data are plotted for the behavioural tasks of Experiment 2. The data shown for the volume oddball task (VO) were calculated across runs.Figure4A_behavioral results is a csv table containing the data used for Figure 4A Rows represent single subjects.Code          			Explanation----          				-----------conflict_effect_RT_CDII      Conflict effect in reaction for content discrimination task IIconflict_effect_ER_CDII     Conflict effect in error rates for content discrimination task IIconflict_effect_RT_LD	 Conflict effect in reaction times for location discrimination taskconflict_effect_ER_LD	 Conflict effect in error rates for location discrimination taskconflict_effect_RT_VO      	 Conflict effect in reaction for volume oddball task conflict_effect_ER_VO     	 Conflict effect in error rates for volume oddball taskconflict_effect_RT_HRDM  Conflict effect in reaction times for horizontal RDM taskconflict_effect_ER_HRDM	 Conflict effect in error rates for horizontal RDM task
