# Supplementary material for: Preserved sensory processing but hampered conflict detection when stimulus input is task-irrelevant
Source: eLife. 2021 Jun 14;10:e64431. doi: 10.7554/eLife.64431 (PMC8294845; doi:10.7554/eLife.64431)
Supplement: Figure 4—figure supplement 1—source data 1. [file elife-64431-fig4-figsupp1-data1.zip › Figure 4 - Supplement 1 /Figure 4 ΓÇô Supplement 1 source data 3.rtf]

Source files for behavioural results of Experiment 2 (related to Figure 4 - Supplement 1)-------------------------------------------------------------In Figure 4 - Supplement 1A-D, the conflict effect (incongruent - congruent) is shown for reaction times (RT), perceptual sensitivity (d-prime), hit rate (HR) and false alarm rate (FAR). These data are plotted for the volume oddball task of Experiment 2. The data is shown for separate runs. Figure 4 - Supplement 1 source data 1 is a csv table containing the raw data used for Figure 4 - Supplement 1A-DRows represent single subjects.Code          			Explanation----          				-----------RT_CE_run1  			Conflict effect in reaction time for run 1 of the volume oddball taskRT_CE_run2			Conflict effect in reaction time for run 2 of the volume oddball taskd_CE_run1  			Conflict effect in d-prime for run 1 of the volume oddball taskd_CE_run2			Conflict effect in d-prime for run 2 of the volume oddball taskhr_CE_run1  			Conflict effect in hit rate for run 1 of the volume oddball taskhr_CE_run2			Conflict effect in hit rate for run 2 of the volume oddball taskfar_CE_run1  			Conflict effect in false alarm rate for run 1 of the volume oddball taskfar_CE_run2			Conflict effect in false alarm rate for run 2 of the volume oddball taskFigure 4 - Supplement 1 source data 2 is a csv table containing the statistical results that are shown in for Figure 4 - Supplement 1A-DFor every run of the volume oddball task, we performed a one sample t-test on the conflict effect (incongruent - congruent) against zero for RT, d-prime, HR and FAR.Code          			Explanation----          				-----------Run					Run number of volume oddball taskdv					dependent variable: either RT, d-prime, HR, or FARt					student-t statisticdf					degrees of freedomp					p-valueCohen’s d				effect size in Cohen’s d	
