# Supplementary material for: Preserved sensory processing but hampered conflict detection when stimulus input is task-irrelevant
Source: eLife. 2021 Jun 14;10:e64431. doi: 10.7554/eLife.64431 (PMC8294845; doi:10.7554/eLife.64431)
Supplement: Figure 5—source data 1. [file elife-64431-fig5-data1.zip › Figure 5/Figure 5 source data 3.rtf]

Source files for decoding results within ROI (related to Figure 5B)-------------------------------------------------------------In Figure 5B, effect sizes (Cohen’s d) of decoding accuracies are plotted for a specific time-frequency ROI, for each task/feature combination. For every task/feature, one-sided, one-sample t-tests were used to test the accuracies against chance-level (x>0.5). Effect sizes were calculated as Cohen’s d.In the following file every column represents a specific task/feature combination:Figure5B_ROI1_accuracies is a csv table containing the data used to perform t-tests and calculation of Cohen’s d for Figure 5B The Cohen’s d scores can be found in:Figure5B_ROI1_cohens_d is a csv table containing the data for Figure 5B Code          	Explanation----          		-----------Task          	Behavioral taskFeature     	Auditory stimulus featuret      			t-value for one-sample t-testdf      		Degrees of freedomCohen’s d 	Effect size (Cohen’s d)
